# Primary Adrenal Failure due to Antiphospholipid Syndrome

**DOI:** 10.1155/2015/161497

**Published:** 2015-10-22

**Authors:** Murat Sahin, Ayten Oguz, Dilek Tuzun, Serife Nur Boysan, Bülent Mese, Hatice Sahin, Kamile Gul

**Affiliations:** ^1^Department of Endocrinology and Metabolism, Faculty of Medicine, Kahramanmaras Sutcu Imam University, 4600 Kahramanmaras, Turkey; ^2^Clinic of Endocrinology and Metabolism, Necip Fazil State Hospital, 46050 Kahramanmaras, Turkey; ^3^Department of Cardiovascular Surgery, Faculty of Medicine, Kahramanmaras Sutcu Imam University, 4600 Kahramanmaras, Turkey; ^4^Clinic of Chest Diseases, Necip Fazil State Hospital, 46050 Kahramanmaras, Turkey

## Abstract

*Background*. Antiphospholipid syndrome (APS) characterized by thrombosis and abortus may rarely cause primary adrenal failure. *Case Presentations*. A 34-year-old male presented with hypotension, hypoglycemia, hyperpigmentation on his skin and oral mucosa, scars on both legs, and loss of consciousness. In laboratory examinations, hyponatremia (135 mmol/L), hyperpotassemia (6 mmol/L), and thrombocytopenia (83 K/*µ*L) were determined. Cortisol (1.91 *µ*g/dL) and adrenocorticotropic (550 pg/mL) hormone levels were also evaluated. The patient was hospitalized with a diagnosis of acute adrenal crisis due to primary adrenal insufficiency. A Doppler ultrasound revealed venous thrombosis. The patient was diagnosed with antiphospholipid syndrome after the detection of venous thrombosis, thrombocytopenia, elevated aPTT, and anticardiolipin antibody levels. Anticoagulation treatment was started for antiphospholipid syndrome. The patient is now following up with hydrocortisone, fludrocortisone, and warfarin sodium. *Conclusion*. Antiphospholipid syndrome is a rare reason for adrenal failure. Antiphospholipid syndrome should be suspected if patients have morbidity secondary to venous-arterial thrombosis.

## 1. Introduction

Primary adrenal failure (PAF) is insufficiency of both mineralocorticoid and glucocorticoid production in the adrenal cortex. The prevalence of primary adrenal failure is 35–60 per million people. The most common reason for PAF is autoimmune adrenal damage (70–90%) [1].

Antiphospholipid syndrome (APS) characterized by thrombosis and abortus rarely causes PAF (<0.5%). APS may occur as an isolated disorder (primary APS) or it may be related to another autoimmune disease (secondary APS). According to the revised Sapporo criteria, APS is considered if at least one of the clinical criteria, such as vascular thrombosis or pregnancy morbidity, and at least one of the laboratory criteria, such as the presence of antiphospholipid antibodies on two or more occasions at least 12 weeks apart, are fulfilled [1, 2].

In this case, a newly diagnosed primary APS presenting with adrenal failure was reported.

## 2. Case

**Figure 1:**
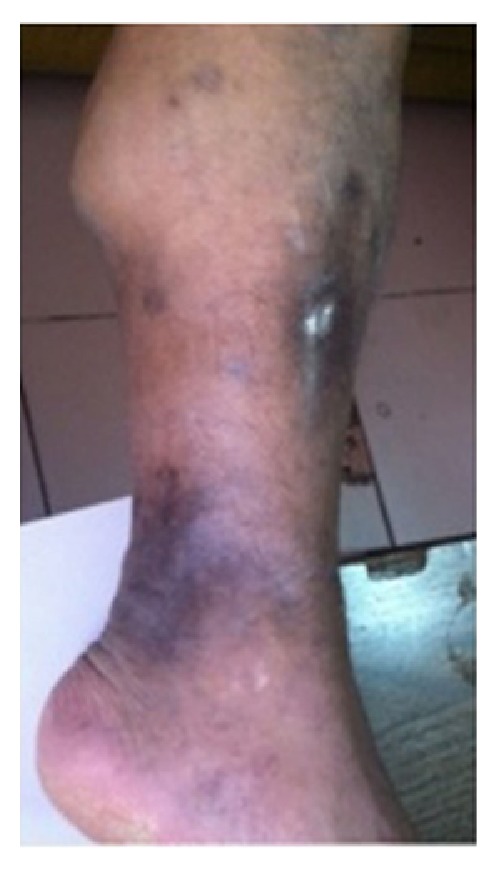
Hyperpigmented scars in extensor and lateral part of leg, 60 × 91 mm.

A 34-year-old male was brought to emergency services due to loss of consciousness. His capillary blood glucose was 37 mg/dL in the ambulance, so 20% dextrose was infused intravenously. At emergency services, his physical examination indicated that he was confused and had a blood pressure of 70/50 mmHg and pulse of 95/min, and temperature was 37°C. His skin and oral mucosa had widespread hyperpigmentation and scars were on both legs (Figure 1). Other laboratory examination results were 1 mg/dL creatinine, 135 mmol/L sodium, 6 mmol/L potassium, 11.7 g/dL hemoglobin, 7.14 K/*μ*L leukocyte count, 83 K/*μ*L thrombocyte count, 5.79 *μ*IU/mL thyroid stimulating hormone, and 1.36 ng/dL free T4. Cortisol and adrenocorticotropic hormone levels (1.91 *μ*g/dL and 550 pg/mL, resp.) were evaluated with the initial diagnosis of adrenal insufficiency due to widespread hyperpigmentation, hypotension, hypoglycemia, and hyperkalemia. Acute adrenal crisis treatment was started and the patient was hospitalized to evaluate the causes of PAF.

He had a history of nausea, vomiting, weakness, fatigue, and skin hyperpigmentation for 1.5 years. He had two presyncope attacks. In addition, he had a medical history of depression and his medications included sertraline (50 mg/day) and olanzapine (5 mg/day).

The patient was evaluated for possible causes of PAF. The magnetic resonance imaging of the adrenal glands was normal. The adrenal hormone measurements (and normal ranges) were 69.8 ng/L (5.3–99.1) direct renin, 40.2 pg/mL (38.1–313.3) aldosterone, 9.27 *μ*g/24 hours (88–444) metanephrine, and 55.03 *μ*g/24 hours (52–341) normetanephrine. He was evaluated for human immunodeficiency virus, syphilis, disseminated fungal infections, and tuberculosis, but no infectious disease was detected. He had no history of drugs causing adrenal insufficiency. He was also evaluated for autoimmune polyglandular syndrome, but the parathormone, C-peptide, calcium, and testosterone levels were normal. No other autoimmune disease accompanied the primary adrenal failure.

**Figure 2:**
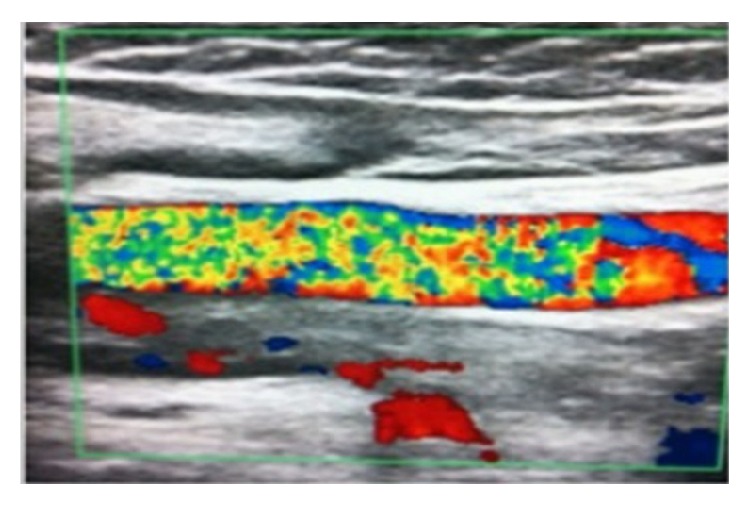
Doppler ultrasound revealed thrombosis in a main femoral vein, 95 × 79 mm.

A bilateral low extremity arterial and venous Doppler ultrasound was performed because of the varicose veins, scars, and stasis ulcers in his legs. The Doppler ultrasound revealed bilateral thromboses in the main femoral vein, right deep femoral vein, superficial femoral vein, popliteal vein, and right vena saphena magna (Figure 2). No thrombus was detected with echocardiography. For thrombosis etiology, blood samples were taken for protein C, protein S, activated partial thromboplastin time (aPTT), and prothrombin time. The results and normal ranges were 85.2% (70–140), 82.4% (60–130), 77.7 seconds (20–35), and 15 seconds (11–16), respectively. Antiphospholipid syndrome was suspected due to the increased aPTT levels and thrombosis. The laboratory test results for antiphospholipid antibodies were 191.2 U/mL (0–15) anticardiolipin IgG, 33.7 U/mL (0–12) anticardiolipin IgM, 14.2 U/mL (0–15) anti-beta-2 glycoprotein IgM, 229.6 U/mL (0–15) anti-beta-2 glycoprotein IgG, and 0.6 U/mL (0–0.8) antinuclear antibody. After 12 weeks, the repeated tests for antiphospholipid antibodies were also high. Based on the revised Sapporo criteria, the patient was diagnosed with primary antiphospholipid syndrome because no disease related to antiphospholipid syndrome was seen. Anticoagulation treatment was started for APS. The patient is now following up with hydrocortisone, fludrocortisone, and warfarin sodium.

## 3. Discussion

The most common cause of primary adrenal failure is the autoimmune destruction of the adrenal glands but infections, metastatic cancers, adrenal hemorrhage, infarct, and medications may cause primary adrenal failure as well. Autoimmune adrenalitis may be isolated or part of an autoimmune polyglandular endocrinopathy. Antibodies against steroidogenic enzymes, especially 21-hydroxylase, are commonly found [1]. In this case, the 21-hydroxylase antibodies were not evaluated, but the patient did not have any autoimmune disease for polyglandular autoimmune syndrome. Previously, tuberculosis was the most common reason for PAF but since tuberculosis has been better controlled, now it accounts for only 7–20 percent of cases [1]. The patient was examined for tuberculosis, but the results were negative.

Conditions associated with hypercoagulation rarely cause adrenal failure; one such disease is antiphospholipid syndrome, which is characterized by antiphospholipid antibodies. Antiphospholipid antibodies affect the coagulation pathway. The characteristic finding in APS is thrombosis with vascular and perivascular inflammation. APS may cause various clinical findings, such as arterial-venous thrombosis, livedo reticularis, cardiac valve involvement, and nephropathy. Other findings may include thrombocytopenia, coagulation test abnormalities, and antiphospholipid antibody positivity, as seen in this case.

Venous thrombosis was observed more frequently than was arterial thrombosis in APS and the most common site for deep vein thrombosis is the veins of the calf, but the hepatic, axillary, and cerebral sinuses may also be involved. The adrenal vein and artery are rarely involved in APS [1, 2]. In this case, bilateral thrombosis was detected in the main femoral vein, right deep femoral vein, superficial femoral vein, popliteal vein, and right vena saphena magna. The venous and calf-site vein involvement were consistent with the literature. For APS diagnosis, at least one clinical finding (one or more episodes of venous, arterial, or small vessel thrombosis and/or morbidity with pregnancy) and at least one laboratory finding (the presence of antiphospholipid antibodies on two or more occasions at least 12 weeks apart) should be present [1, 2]. The case was diagnosed as primary antiphospholipid syndrome with thrombosis and antiphospholipid antibody positivity after other reasons for antiphospholipid syndrome were excluded.

Many organs, as well as the adrenal glands, may be involved in antiphospholipid syndrome. In a study evaluating 1000 patients with either primary or secondary APS, common clinical manifestations were deep vein thrombosis (38.9%), thrombocytopenia (29.6%) livedo reticularis (24.1%), stroke (19.8%), pulmonary embolism (14.1%), and hemolytic anemia (9.7%) with only 4 patients having primary adrenal failure (0.4%) [2]. In the present case, the patient had common clinical manifestations, such as deep vein thrombosis and thrombocytopenia. Primary adrenal failure in APS is probably related to spontaneous hemorrhagic infarct or hemorrhagic necrosis due to adrenal vein thrombosis [3, 4]. The vascularity of adrenal glands is important in pathogenesis; adrenal glands have a rich arterial supply but a limited venous drainage, and this feature may be predisposing to thrombosis [5, 6].

Adrenal glands have a high cholesterol content and cells with high lipid trafficking are rich in lysobisphosphatidic acid, which are targeted by antiphospholipid antibodies [6]. Another possible mechanism suggests that antiphospholipid antibodies increase intracellular cholesterol deposition, causing cell death [2, 7]. Even though APS is more common in females, adrenal involvement in APS is more frequent in males (55%), and adrenal failure is the initial finding of APS in 36% of patients [8]. Consistent with the literature, the patient was male and adrenal failure was the initial finding of APS. Satta et al. presented a case with adrenal insufficiency as the first clinical manifestation of primary antiphospholipid antibody syndrome. The patient's adrenal imaging was normal, as in the present case, so they speculated that intraparenchymal microhemorrhages might not be detectable with radiologic imaging [9].

PAF may present with nonspecific findings, such as nausea, vomiting, fatigue, anorexia, weakness, hypotension, and hypoglycemia similar to gastrointestinal and psychiatric diseases. Chronic adrenal insufficiency may cause psychiatric symptoms and psychosis [10], as in the current case. Antiphospholipid syndrome is a rare cause of adrenal insufficiency. Antiphospholipid syndrome should be suspected if patients have morbidity secondary to venous-arterial thrombosis.
